# Targeted Therapy in Systemic Sclerosis

**DOI:** 10.5041/RMMJ.10257

**Published:** 2016-10-31

**Authors:** Murray Baron

**Affiliations:** Chief Division of Rheumatology, Jewish General Hospital, Montreal, Quebec, Canada; and Professor of Medicine, McGill University, Montreal, Quebec, Canada

**Keywords:** Drug treatment, scleroderma, systemic sclerosis, targeted, therapy

## Abstract

Targeted therapies use an understanding of the pathophysiology of a disease in an individual patient. Although targeted therapy for systemic sclerosis (SSc, scleroderma) has not yet reached the level of patient-specific treatments, recent developments in the understanding of the global pathophysiology of the disease have led to new treatments based on the cells and pathways that have been shown to be involved in the disease pathogenesis. The presence of a B cell signature in skin biopsies has led to the trial of rituximab, an anti-CD20 antibody, in SSc. The well-known properties of transforming growth factor (TGF)-β in promoting collagen synthesis and secretion has led to a small trial of fresolimumab, a human IgG4 monoclonal antibody capable of neutralizing TGF-β. Evidence supporting important roles for interleukin-6 in the pathogenesis of SSc have led to a large trial of tocilizumab in SSc. Soluble guanylate cyclase (sGC) is an enzyme that catalyzes the production of cyclic guanosine monophosphate (cGMP) upon binding of nitric oxide (NO) to the sGC molecule. Processes such as cell growth and proliferation are regulated by cGMP. Evidence that sGC may play a role in SSc has led to a trial of riociguat, a molecule that sensitizes sGC to endogenous NO. Tyrosine kinases (TKs) are involved in a wide variety of physiologic and pathological processes including vascular remodeling and fibrogenesis such as occurs in SSc. This has led to a trial of nintedanib, a next-generation tyrosine-kinase (TK) inhibitor which targets multiple TKs, in SSc.

## INTRODUCTION

Systemic sclerosis (SSc, or scleroderma) is a serious multi-system disorder of connective tissue disease characterized clinically by thickening and fibrosis of the skin and involvement of internal organs, most commonly the lungs, gastrointestinal tract, and heart. Systemic sclerosis affects predominantly women in the prime of their life and is associated with increased morbidity and mortality. Systemic sclerosis is a rare disease with prevalence estimates varying from 30 to 443/million population.^1^,^2^

Because the disease is so heterogeneous and rare, it has been difficult to perform high-quality randomized controlled clinical trials. Unfortunately, the few such trials performed, especially early ones, have not been uniformly successful. Therapies studied in randomized controlled trials that have failed include ketanserin,^3^ 5-fluorouracil,^4^ ketotifen,^5^ interferon-gamma,^6^ interferon-alpha,^7^ penicillamine,^8^ relaxin,^9^,^10^ methotrexate,^11^ oral collagen,^12^ imatinib,^13^,^14^ IVIG,^15^ macitentan,^16^ and bosentan.^17^,^18^ More recently there have been some hopeful positive trials with statins,^19^ cyclophosphamide,^20^ and autologous stem cell transplantation.^21^,^22^

Could we be more successful with a more targeted approach to therapy in SSc, similar to that taken in other diseases such as cancer? For example, in melanoma, BRAF kinase-activating mutations can turn BRAF into an oncogene, and the presence of such mutations has led to specific therapy.^23^ Similar analyses of non-small cell lung cancer has found that mutations in the epidermal growth factor receptor (EGFR), translocations involving the anaplastic lymphoma kinase (ALK) tyrosine kinase, oncogenic RAS mutations, and other driver mutations, such as BRAF, can be treated with therapy targeted at these abnormalities.^24^

In SSc we are entering a very exciting and hopeful era with 316 trials for SSc listed on ClinicalTrials.gov.^25^ Some of these trials have used, or are using, new biologic agents or kinase inhibitors such as abatacept,^26^ rituximab,^25^ imatinib, dasatinib, fasudil,^27^ nintedanib,^28^ and fresolimumab, an anti-transforming growth factor (TGF) antibody.^29^ A phase III trial of tocilizumab is underway. Some of these trials are based on the concept of targeting specific pathophysiologic abnormalities that have been found in SSc. We have thus hopefully moved away from general immunosuppressives such as cyclophosphamide or methotrexate to drugs that inhibit specific pathways or cells thought to be active in SSc. These represent the first steps toward personalized targeted medicine in SSc.

## RITUXIMAB

One promising approach to possible biomarkers that may indicate that a certain subset may respond to specific targeted therapy has come from the study of the gene expression found in skin biopsies.^29^–^37^ Early work demonstrated that some patients with SSc had a skin gene expression signature consistent with the presence of active B cells.^31^ This observation has led directly to two trials of rituximab (RTX), an anti-CD20 monoclonal antibody, in SSc.^38^,^39^ In the first publication 15 patients with diffuse cutaneous systemic sclerosis (dcSSc) received two doses of rituximab 1 g each, 2 weeks apart.^39^ The mean change in the modified Rodnan skin score (MRSS) between baseline and 6 months was not significant. Results of pulmonary function tests and other measures of major organ involvement were stable. The modest B cell infiltrates that were present in most skin biopsy specimens at baseline were completely depleted at 6 months in most patients. In the other trial, 14 patients with SSc were evaluated.^38^ Eight patients were randomized to receive two cycles of RTX at baseline and 24 weeks, whereas six patients (control group) received standard treatment alone. There was a significant increase of forced vital capacity (FVC) in the RTX group compared with baseline. The median percentage of improvement of FVC in the RTX group was 10.25%, whereas that of deterioration in the controls was 5.04% (*P*=0.002). Skin thickening, assessed with the MRSS, improved significantly in the RTX group compared with the baseline score (mean±SD 13.5±6.84 versus 8.37±6.45 at baseline versus 1 year, respectively, *P*<0.001). Rituximab depleted both circulating B cells and dermal B cells. In the RTX-treated group, there was a significant reduction of collagen deposition in the papillary dermis at 24 weeks compared with baseline which was not seen in the control group. The EUSTAR group also published an observational study assessing the effects of RTX on skin and lung fibrosis in patients with SSc.^40^ Comparison between RTX-treated patients and matched controls revealed a significant difference in favor of RTX. However, to this author’s knowledge, rituximab has not strictly been employed as targeted therapy such as treating only those patients with a skin biopsy showing B cell activation or analyzing the results in patients with skin biopsies that show B cell activation versus those with no such pattern.

## FRESOLIMUMAB

It is well-known that TGF-β promotes collagen synthesis, secretion, processing, and cross-linking as well as secretion of other matrix molecules, such as fibronectin and thrombospondin.^41^ This has led to an interest in using anti-TGF-β in SSc. The first trial of CAT-192, a recombinant human antibody that neutralizes TGF-β1, was not successful.^42^ However, a more recent study tested fresolimumab, a first-in-class human IgG4 κ monoclonal antibody capable of neutralizing all mammalian isoforms of TGF-β.^29^ There is a four-gene, pharmacodynamic biomarker of SSc skin disease, based on gene expression in a mid-forearm skin biopsy.^29^ Two of the four genes making up the biomarker, thrombospondin-1 (*THBS1*) and cartilage oligomeric protein (*COMP*), are highly regulated by TGF-β. In this open uncontrolled trial, the predefined primary efficacy outcome was change in *COMP* and *THBS1* mRNA expression in skin after treatment compared with that at baseline. Subjects showed rapid declines in *THBS1* and *COMP* gene expression in skin biopsies after treatment with fresolimumab. *THBS1* and *COMP* gene expression was strikingly higher in SSc patient cohorts than in healthy control skin, and changes in gene expression in study patients generally correlated with changes in MRSS.

## TOCILIZUMAB

Another promising therapy consists of inhibition of interleukin (IL)-6. Evidence supports important roles for IL-6 in the pathogenesis of SSc, e.g. dermal fibroblasts from SSc patients constitutively express higher levels of IL-6 than found in healthy controls; serum and skin levels of IL-6 are elevated in SSc patients with early disease and in patients with SSc or SSc-interstitial lung disease (ILD), and increased IL-6 levels have been associated with higher mortality, more severe skin involvement, and increased incidence of progressive pulmonary decline.^43^–^48^ Strategies to block the IL-6 response resulted in a significant reduction of procollagen type I in cultured SSc fibroblasts and myofibroblastic differentiation in dermal fibroblasts in a bleomycin-induced model of dermal sclerosis.^49^,^50^ Some indirect evidence of increased effect of IL-6 in SSc derives from the fact that CRP is elevated in SSc although not to levels associated with diseases such as rheumatoid arthritis.^51^ C-reactive protein (CRP) levels in dcSSc are higher than in limited cutaneous SSc (lcSSc).^51^,^52^

These findings have led to a phase II trial of tocilizumab in early dcSSc.^53^ The primary end-point showed a treatment difference of −2.70 MRSS units in favor of tocilizumab at week 24 but did not quite reach statistical significance. Exploratory analysis of lung function showed that fewer patients in the tocilizumab arm had a decline in percentage-predicted forced vital capacity than in the placebo arm by comparison of the cumulative distribution by week 48. Tocilizumab specifically downregulated the expression of myeloid-associated genes in the skin and decreased circulating levels of CCL18, a chemokine associated with fibrosis and progression of SSc-associated lung disease. A phase III trial is underway.

## RIOCIGUAT

Soluble guanylate cyclase (sGC) is an enzyme that catalyzes the production of cyclic guanosine monophosphate (cGMP) upon binding of nitric oxide (NO) to the sGC molecule. Once released by the sGC, cGMP can act as a second messenger to activate further downstream targets, such as cGMP-regulated ion channels, protein kinases (G-kinases), and phosphodiesterases (PDEs). Through those effectors, cGMP regulates a variety of physiological processes, including cell growth and proliferation, vascular tone and remodeling, immune responses, and neuronal transmission. Riociguat is a molecule that sensitizes sGC to endogenous NO by stabilizing NO–sGC binding.^54^,^55^ Riociguat also directly stimulates sGC, independent of NO, resulting in increased generation of cGMP. Riociguat has been shown in large randomized controlled clinical trials to be effective in patients with different forms of pulmonary hypertension including patients with SSc-related pulmonary arterial hypertension (PAH).

There is evidence that sGC may play a role in SSc. Soluble guanylate cyclase activators inhibited the release of TGF-β-induced extracellular matrix proteins from primary dermal fibroblasts obtained from both normal volunteers and SSc subjects, and dermal fibrosis was reduced in the bleomycin skin fibrosis model of SSc.^56^,^57^ Riociguat has been shown in large randomized controlled clinical trials to be effective in patients with different forms of pulmonary hypertension, including patients with SSc-related PAH,^58^ and is now in a trial for the skin thickening of SSc.

## NINTEDANIB

The last molecule that we will briefly address is nintedanib, a next-generation, potent, indolinone-derived small molecule tyrosine-kinase (TK) inhibitor which targets multiple TKs.^59^–^61^ Tyrosine kinases are involved in a wide variety of physiologic and pathological processes including vascular remodeling and fibrogenesis. Nintedanib leads to inhibition of several central molecules involved in fibroblast activation such as PDFGR-α and PDFGR-β, FGFR-1, FGFR-2, FGFR-3, VEGFR-1, VEGFR-2, VEGFR-3, and Src.

Nintedanib reduced differentiation of myofibroblasts and the release of collagen of dermal fibroblasts from patients with SSc and healthy individuals. Nintedanib also showed anti-fibrotic effects in a dose-dependent manner in different animal models of SSc, including the bleomycin skin fibrosis model both in preventive and therapeutic applications, the chronic graft-versus-host disease model, and the Tsk-1 model. Interestingly, in the Fra-2 tg mouse model, nintedanib did not only inhibit skin and lung fibrosis but also improved the pulmonary vascular lesions resembling PAH.^61^ Based on these results, a large, randomized, placebo-controlled trial with nintedanib is currently initiated in patients with SSc for pulmonary fibrosis.

## CONCLUSION

Although we are in an exciting new era of drug trials for SSc, and although the drugs discussed here are being used because of their abilities to interfere with specific cells or pathways that have been found to be involved in SSc or SSc models, the field of targeted therapy has not yet progressed quite as far as it has in cancer. The next big step will be to understand if the new drugs are effective specifically in patients who demonstrate abnormalities of the cells or pathways that are inhibited or activated by these new medications. Then we will be closer to developing a truly personalized approach to the treatment of this serious disease.

## Abbreviations

ALK: anaplastic lymphoma kinase
cGMP: cyclic guanosine monophosphate
COMP: cartilage oligomeric protein
dcSSc: diffuse cutaneous systemic sclerosis
EGFR: epidermal growth factor receptor
FVC: forced vital capacity
G-kinases: protein kinases
GMP: guanosine monophosphate
IL-6: interleukin-6
ILD: interstitial lung disease
lcSSc: limited cutaneous systemic sclerosis
MRSS: modified Rodnan skin score
NO: nitric oxide
PDEs: phosphodiesterases
RTX: rituximab
sGC: soluble guanylate cyclase
SSc: systemic sclerosis, scleroderma
TGF-β: transforming growth factor beta
THBS1: thrombospondin-1
TK: tyrosine kinase
